# The Synergistic Activity of Bortezomib and TIC10 against A2058 Melanoma Cells

**DOI:** 10.3390/ph14080820

**Published:** 2021-08-20

**Authors:** Angéla Takács, Zsófia Szász, Márton Kalabay, Péter Bárány, Antal Csámpai, Hargita Hegyesi, Orsolya Láng, Eszter Lajkó, László Kőhidai

**Affiliations:** 1 Department of Genetics, Cell and Immunobiology, Semmelweis University, H-1089 Budapest, Hungary; angela.takacs1@gmail.com (A.T.); szaszzsoccii@gmail.com (Z.S.); martonkalabay@gmail.com (M.K.); hegyesi.hargita@med.semmelweis-univ.hu (H.H.); langorsi@gmail.com (O.L.); lajesz@gmail.com (E.L.); 2 Department of Organic Chemistry, Institute of Chemistry, Eötvös Loránd University, H-1117 Budapest, Hungary; baranypeti87@gmail.com (P.B.); csampai@caesar.elte.hu (A.C.)

**Keywords:** combination therapy, bortezomib, TIC10, antitumor efficacy, melanoma

## Abstract

Combination antitumor treatments are essential parts of modern tumor therapy as—compared to monotherapies—(i) they are more effective; (ii) the dose of the compounds can be reduced; and (iii) therefore the side effects are improved. Our research group previously demonstrated the antitumor character of bortezomib (BOZ) in A2058 melanoma cells. Unfortunately, dose-related side effects are common during BOZ therapy, which could be prevented by reducing the dose of BOZ. This study aimed to characterize synergistic combinations of BOZ with a TRAIL (TNF-related apoptosis-inducing ligand) -inducing compound (TIC10), where the doses can be cut down but the efficacy is preserved. Endpoint cell viability assays were performed on A2058 cells, and synergism of BOZ and TIC10 was observed after 72 h. Synergism was further validated in a real-time impedimetric assay, and our results showed that BOZ-treated melanoma cells survived the treatment, an effect not registered in the co-treatments. Treatment with the combinations resulted in increased apoptosis, which was not accompanied by enhanced LDH release. Nevertheless, the expression of death receptor 5 (DR5) was increased on the cell surface without transcriptional regulation. In summary, our findings support the theory that the application of BOZ and TIC10 in combination could provide higher efficacy in vitro.

## 1. Introduction

Melanoma is a cancer of pigment-producing melanocytes that can be found in the skin [1,2]. However, we already know what risk factors can lead to the formation of melanoma such as excessive exposure to ultraviolet (UV) radiation [3] and what exact mechanisms can be observed, such as DNA alterations and somatic driver mutations [1]. Unfortunately, the morbidity rates are still increasing [4], and 80% of skin-related deaths are associated with melanoma [5]. Both the surgical removal of the localized tumor and systematic treatments are important in the therapy. The main options to increase the efficacy of melanoma treatment belong to the groups of adjuvant targeted therapies (e.g., mitogen-activated protein kinases (MAPK) pathway inhibitors) or immunotherapies (e.g., immune checkpoint inhibitors) [6,7]. These options could increase the overall survival rates, although the development of drug resistance is an emerging issue [7]. To overcome resistance, targeting multiple cellular pathways with combination treatments may be effective [8]. Combinations can also result in greater clinical activity compared to the clinical activity of the individual drugs. Furthermore, during combination therapies, the dose of the individual compounds can be cut down, and thus drug-associated side effects and off-target effects may be reduced. Due to these advantages, the survival rates, as well as the quality of life of the treated patients, can be improved [9].

In our previous study, it was found that bortezomib (BOZ) can have an antitumor effect in A2058 melanoma cells [10]. Clinical trials also suggested that the administration of BOZ is safe for melanoma patients [11]. However, the proteasome inhibitor BOZ may cause severe side effects such as bortezomib-induced peripheral neuropathy (BIPN), and thus dose reduction or even discontinuation can be needed [12,13]. 

Earlier, the antitumor effects of several small molecules had been investigated against A2058 cells, particularly the TRAIL (TNF-related apoptosis-inducing ligand)-inducing compound TIC10 [14]. Possible antitumor effects can be linked to TIC10: (i) it can selectively antagonize dopamine receptor D2, (ii) inactivate the intracellular Akt/ERK pathway, (iii) induce an ER stress response and (iv) upregulate the expression of TNF-related apoptosis-inducing ligand (TRAIL) [15,16,17]. TRAIL can initiate extrinsic apoptosis through binding its receptors (death receptor 4 (DR4) and death receptor 5 (DR5)) [18]. Through a downstream intracellular signaling pathway, namely, the formation of death-inducing signaling complex (DISC), activator and effector caspases are activated that can be responsible for the induction of apoptosis [19]. Despite these mechanisms, no IC_50_ value could be determined after 72 h against the A2058 melanoma cell line in our previous study [14]. 

In the literature, it was demonstrated that BOZ could enhance the expression of proteins, e.g., DR4 or DR5, in HT-29 human colorectal adenocarcinoma cells, in A549 human lung adenocarcinoma cells and in human non-small cell lung cancer cells [20,21]. We hypothesized that following a co-treatment with BOZ and TIC10, the enhancement in the expression of the TRAIL protein post-TIC10 treatment and the upregulation of the death receptors post-BOZ treatment could lead to reduced cell viability. This gave us the idea to find a possible synergistic combination of BOZ with the small molecule TIC10.

In this study, we aimed to define a synergistic combination of BOZ with TIC10 in A2058 melanoma cells and then to characterize the underlying synergistic antitumor mechanisms of this combination compared to the effects of individual compounds.

## 2. Results

### 2.1. Influence of the Compounds on Cell Viability and Combination Index Assessments

**Figure 1:**
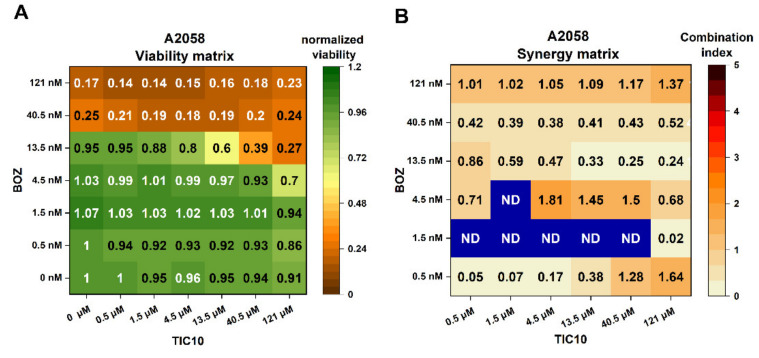
Heat maps showing antiproliferative effects of bortezomib (BOZ), TIC10 and their combinations on A2058 cells after 72 h of incubation. (**A**) Normalized viability data are expressed as a ratio of medium control, and (**B**) combination index heat maps of various drug ratios. Combination index < 1, = 1 or > 1 represents synergism, additive effect or antagonism, respectively. ND: not detectable. Data are presented as mean values (*n* = 3).

In this study, at first, two different cell lines, A2058 melanoma and U266 myeloma, were used to determine the antiproliferative effects of bortezomib (BOZ; 0.5–121 nM), TIC10 (0.5–121 μM) and the varying ratios of the BOZ+TIC10 co-treatments. These endpoint cell viability assays were carried out after 72 h treatments because, according to the literature, TIC10 induces apoptosis through the induction of the transcription of the TRAIL gene and can take effect only after a longer incubation time [22,23]. Table 1 summarizes the IC_50_ values of the single agents on A2058 and U266 cells. Bortezomib showed dose-dependent antiproliferative effects on both cell lines (Figure 1A and Figure S1A, Supplementary Materials); however, the myeloma cell line was more sensitive than the melanoma cell line (1.45 ± 0.06 nM and 23.07 ± 2.41 nM, respectively). TIC10 had an antitumor effect only on U266 cells, but not on A2058 cells. The solvent dimethyl sulfoxide (DMSO) could influence the U266 cell viability, but this was not observed in the A2058 cells (Table S1, Supplementary Materials). In the same experiment, the effects of the BOZ+TIC10 combinations were analyzed (Figure 1A and Figure S1A, Supplementary Materials), and then—based on these results—combination index (CI) analyses were performed, and synergy matrices were created (Figure 1B and Figure S1B, Supplementary Materials). The U266 cells were very sensitive to BOZ, and in the presence of 4.5 nM BOZ, the normalized cell viability was only 0.01 (1%). In A2058 cells, the synergistic effects were more significant than in the U266 cells. Combination index values less than 1 indicate a synergism between BOZ and TIC10. The synergy matrix of A2058 shows that 4.5 nM BOZ was the first concentration where synergism could be detected in combination with TIC10. The criteria for the combinations, which were analyzed in the further experiments, were the following: (i) where the decrease in the cell viability was around 50% or more compared to the matching single-agent treatment, (ii) where the concentrations of the single agents were as low as possible and (iii) where the DMSO was under 0.5 *v/v*%. With the help of these criteria, two combinations, 13.5 nM BOZ + 40.5 μM TIC10 and 13.5 nM BOZ + 13.5 μM TIC10 (CI = 0.25 and 0.33, respectively), were identified in A2058 cells. These combinations were selected for the subsequent experiments; in addition to these, the matching single agents and controls (medium and DMSO) were also examined. Probably due to the high sensitivity of U266 cells to BOZ, its combinations with TIC10 could not improve the cell viability decreasing effect. Therefore, this cell line was excluded in the following experiments.

**Figure 2:**
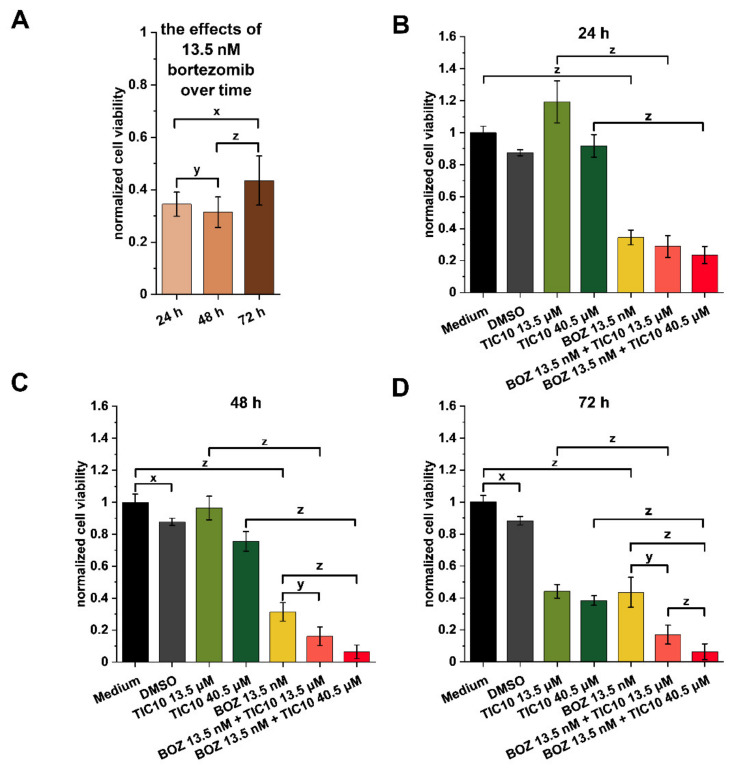
Analysis of cell viability at different time points of treatment. A real-time assay was conducted to observe changes over time (**A**) after bortezomib (BOZ) treatment and (**B**) after 24 h, (**C**) after 48 h and (**D**) after 72 h treatments with the combinations. The data were normalized to the medium control. Data are presented as mean values ± standard deviation (SD) (*n* = 3). The levels of significance are shown as follows: x: *p* < 0.05; y: *p* < 0.01; z: *p* < 0.001, determined by a one-way ANOVA test followed by Fisher’s LSD post hoc test.

To prove the results of the endpoint cell viability assay, a real-time cell viability assay (xCELLigence SP System, ACEA Biosciences, San Diego, CA, USA) was performed on A2058 cells, where 13.5 nM BOZ + 13.5 μM TIC10, 13.5 nM BOZ + 40.5 μM TIC10 and the matching single agents and controls (medium and DMSO) were used. Although 13.5 nM BOZ alone could drastically affect the cell viability (Figure 2A), after 48 h, a population of the melanoma cells could start to proliferate, and the measured cell index started to increase (Figure S2, Supplementary Materials). This developed to its maximum in the 72nd hour of the treatment (Figure 2A and Figure S2, Supplementary Materials). The used TIC10 concentrations started to take effect after 48 h, but the most significant effects were registered after 72 h (Figure 2B–D and Figure S2, Supplementary Materials). The results of the co-treatments also show that the maximum effects were reached at the end of the 72 h treatment (Figure 2B–D); thus, the following experiments were conducted after 72 h.

### 2.2. Effects on Cell Death and Cytotoxicity

**Figure 3:**
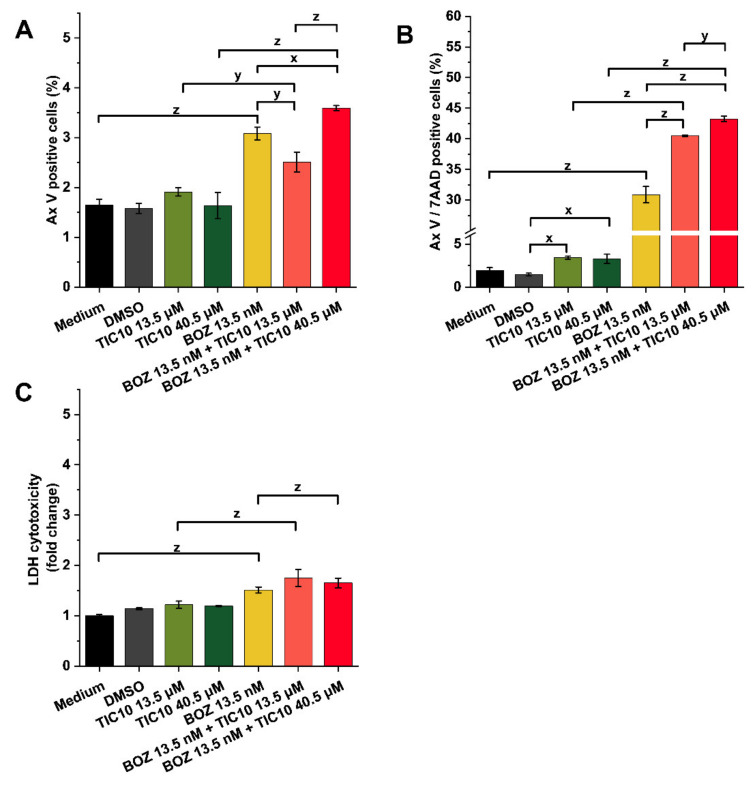
Analysis of the apoptotic and cytotoxic effects of the treatments. (**A**) Percentage of early apoptotic (Ax V-positive) and (**B**) late apoptotic (Ax V and 7AAD double-positive) cells, and (**C**) the fold change of the LDH release after 72 h treatment with bortezomib (BOZ), TIC10 and their combinations. (**C**) The data were normalized to the medium control. Data are presented as mean values ± standard deviation (SD) (*n* = 3). The levels of significance are shown as follows: x: *p* < 0.05; y: *p* < 0.01; z: *p* < 0.001, determined by a one-way ANOVA test followed by Fisher’s LSD post hoc test.

Annexin V-fluorescein isothiocyanate (Ax V-FITC) and 7-aminoactinomycin (7-AAD) staining was conducted in an attempt to compare the cell death-inducing effects of the different treatments. Figure 3 details the data on cell death (early and late apoptosis) and cytotoxicity. The solvent DMSO did not affect apoptosis compared to the untreated medium control. Furthermore, neither 13.5 μM nor 40.5 μM TIC10 could significantly affect the number of Ax V-positive, early apoptotic cells (Figure 3A), and the double-positive late apoptotic cells (Figure 3B). In contrast to this, BOZ was capable of significantly increasing the number of Ax V-positive cells (Figure 3A), although the increase in the number of Ax V/7-AAD double-positive cells was more remarkable (Figure 3B). Exposure to the combinations induced a significant increase in cell death, including early (Ax V positive cells) and late apoptosis (Ax V/7-AAD double-positive cells), compared to the cells exposed to the single agents (Figure 3A,B, respectively).

The LDH release assay revealed that BOZ and the co-treatments did not affect the level of LDH release of the A2058 cells (Figure 3C). Light microscopic analysis indicated that cells after 13.5 nM BOZ treatment perished, but TIC10 treatments rather had growth-inhibitory effects (Figure S3, Supplementary Materials).

### 2.3. Association of Synergism with Death Receptor Expression at mRNA and Protein Level

Table 2 compares the data obtained from the experiments on the mRNA expression levels of death receptor 4 (DR4) and death receptor 5 (DR5). Unexpectedly, TIC10 treatments caused a decreased mRNA level of both death receptors. The reduced DR5 receptor expression was also observed in BOZ-treated and BOZ+TIC10-treated cells after 72 h. On the other hand, the treatment of A2058 cells with BOZ and with co-treatments resulted in a slight overexpression of DR4 receptors. 

The mRNA expression of death receptor 4 (DR4) and death receptor 5 (DR5) was normalized to the geometric mean of the mRNA levels of GAPDH and HPRT1 and calculated by the 2^(−∆∆Ct)^ method. Expression values are relative to the medium control.

**Figure 4:**
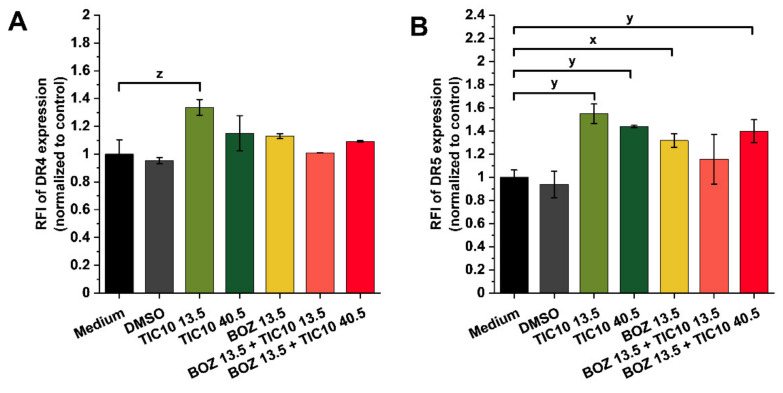
Influence on the death receptor 4 (DR4) and death receptor 5 (DR5) expression of melanoma cell line after 72 h exposure. The ratio of the mean fluorescence intensity (RFI) of (**A**) DR4 expression and (**B**) DR5 expression is reported. The data were normalized to the medium control (RFI = treated cells MFI/control cells MFI; MFI: mean fluorescence intensity). Data are presented as mean values ± standard deviation (SD) (*n* = 2). The levels of significance are shown as follows: x: *p* < 0.05; y: *p* < 0.01; z: *p* < 0.001, determined by a one-way ANOVA test followed by Fisher’s LSD post hoc test.

Figure 4 details the data on the possible changes in the DR4 and DR5 protein expression after 72 h treatments with BOZ, TIC10 and their combinations. It is visible in Figure 4A that only 13.5 μM TIC10 had an impact on the DR4 expression of the melanoma cells. At the same time, significant differences were observed in DR5 expression. An increase was measured after 13.5 μM TIC10, 40.5 μM TIC10, 13.5 nM BOZ and 13.5 nM BOZ + 40.5 μM TIC10 treatments (Figure 4B). However, there was no significant difference between the effects of the single treatments and their combinations. 

## 3. Discussion

Our study provides the framework for a new possible synergistic combination therapy with BOZ and TIC10. In vitro viability tests were performed in two different cell lines (A2058, melanoma; U266, multiple myeloma) to define the synergistic activity between the two compounds, and the results were later confirmed with further assays.

According to the results of the cell viability assays, differences in the sensitivity of the cells against the two compounds were detected. Bortezomib is a part of the therapy of myeloma patients [24], but according to the literature, it could be administered to patients suffering from solid tumors, too [25]. The U266 myeloma cells were more sensitive to BOZ compared to the A2058 melanoma cells (IC_50_ values: 1.45 nM and 23.07 nM, respectively), and TIC10 showed inhibitory effects only on U266 cells (IC_50_ value: 0.97 μM). Due to the high sensitivity of the U266 myeloma cells, no synergistic combination of BOZ and TIC10 could be detected; thus, this cell line was excluded from the further assays. In the case of the A2058 melanoma cells, three combinations (13.5 nM BOZ + 13.5 μM TIC10; 13.5 nM BOZ + 40.5 μM TIC10; 13.5 nM BOZ + 121 μM TIC10) were identified by the combination index calculation in the CompuSyn software, where the cell viability results of the combinational therapies are promising and are below the results of the matching monotherapies. According to the literature, the detected combination index values (CI = 0.33, 0.25 and 0.24, respectively) indicate synergism and strong synergism [26]. In this study, 1.21 *v/v*% DMSO decreased the cell viability of U266 cells but had no impact on the A2058 cells. Similar effects of DMSO were reported earlier by Chen et al. [27]. In our studies, 121 μM TIC10, its combinations with BOZ and the matching DMSO control (1.21 *v/v*%) were characterized in the cell viability assay and proved to result in a low viability of the target cells. Nevertheless, these combinations were not investigated in the following experiments due to the high concentration of DMSO in these solutions.

Next, an additional cell viability test was performed on the A2058 cells in the xCELLigence SP system to prove the results of the endpoint cell viability assay. This method is based on the measurement of the changes in impedance over time and has a crucial advantage because it can monitor the changes in the number of cells in real time [28]. This test revealed that a subpopulation of the A2058 cells had lost their sensitivity to BOZ, and despite the 13.5 nM BOZ treatment, an increase in the number of viable cells was detectable. This result indicates that the A2058 melanoma cells might develop acquired resistance to BOZ, which has already been reported in other cell lines (e.g., non-small cell lung carcinoma) and in clinical results (e.g., kappa light chain multiple myeloma patient treated with bortezomib), too [29,30,31]. Interestingly, no increase in the cell viability was observed in cells treated by the combination of BOZ+TIC10. Our result is consistent with previous results, where chemotherapy-resistant cells (e.g., dexamethasone-resistant multiple myeloma cells, bortezomib-resistant ANBL-6 cells) were sensitive to TIC10 [32,33].

In our experiments, the bortezomib-induced decrease in cell viability was enhanced by TIC10. The results were also proved by an Ax V/7AAD apoptosis assay, where the percentage of the double-positive cells after treatments with the combinations was increased compared to the BOZ- or TIC10-treated cells. However, the monoagent TIC10 affected neither the cell viability nor the number of Ax V/7AAD-positive cells compared to the control. The brightfield images also revealed the different responsiveness of the A2058 cells to BOZ and TIC10. These trends were accompanied by a negligible increase in the LDH release. In contrast with these outcomes, these effects were accompanied by a negligible increase in the LDH release. These observations together indicate that BOZ could initiate direct cell death in A2058 cells, whereas TIC10 showed antiproliferative properties and is capable of inhibiting the growth of the cells.

Many studies related to TIC10 mentioned that it can upregulate the TRAIL expression of the tumor cells, and therefore it can selectively induce TRAIL-mediated apoptosis of the cancer cells [34,35,36]. The selectivity of the cancer cells to TIC10 and TRAIL-inducing compounds relies on the overexpression of DR4 and DR5 compared to non-cancerous cells [37,38]; thus, these compounds are promising targeted drugs. In earlier studies, it was reported that BOZ could enhance the expression of these death receptors (DR4 and DR5) [20,21,39]. As it was proposed, our results confirm these previous findings as increased surface receptor expression of DR5 was detected after BOZ and BOZ+TIC10 treatments in the investigated A2058 melanoma cell line. Interestingly, TIC10 itself had an enhancing effect on the expression of both death receptors, too. To further clarify the role of the treatments on protein expression, their modulation on the mRNA level was examined by real-time PCR. These results stand in contrast with the findings on protein levels, as it was found that expression of the DR4 receptor—instead of DR5—was transcriptionally induced in BOZ and co-treated cells. This suggests that BOZ-induced overexpression of the DR5 protein on the surface of the A2058 melanoma cells is not regulated transcriptionally. Further data collection would be required to reveal more details on the exact mechanisms of how the TIC10-induced increased level of the TRAIL protein [22] with the BOZ-induced upregulation of the DR5 protein can result in synergism.

## 4. Materials and Methods

### 4.1. Substances

Bortezomib (Velcade^®^ 3.5 mg; Janssen-Cilag GmBH, Neuss, Germany) was solubilized in distilled water (2 × 10^−3^ M). TIC10 was obtained from Merck/Sigma-Aldrich^®^ (Darmstadt, Germany) and kindly provided by Prof. Csámpai (Institute of Chemistry, Eötvös Loránd University, Budapest, Hungary). It was solubilized in dimethyl sulfoxide (DMSO; AppliChem GmbH, Darmstadt, Germany) (stock solution: 10^−2^ M). The *v/v*% of DMSO must be kept under 0.5 [27]. In each case, fresh solutions were prepared for each experiment.

### 4.2. Cell Culturing

The two investigated cell lines were a human melanoma (A2058) and a human myeloma (U266) cell line, which were obtained from the European Collection of Authenticated Cell Cultures (ECACC, Salisbury, UK).

The A2058 cell line used in this study is an adherent metastatic melanoma culture (91100402 ECACC), while U266 cells (85051003 ECACC) are grown in suspension. Both cell lines were cultured in RPMI 1640 medium (Sigma Ltd., St. Louis, MO, USA) which was supplemented with 10% fetal bovine serum (Invitrogen Corporation, New York, NY, USA), 1% L-glutamine (Invitrogen Corporation, New York, NY, USA) and 1% penicillin/streptomycin (Invitrogen Corporation, New York, NY, USA). The A2058 cells were passaged twice weekly and split 1:3 to 1:6. The medium of U266 was also removed twice a week and then replaced with a fresh medium.

### 4.3. Endpoint Viability Assay

To measure the antiproliferative effects of BOZ, TIC10 and their combinations in A2058 cells, the alamarBlue^TM^ (Thermo Scientific, Waltham, MA, USA) cell viability reagent was used. Cells were seeded in a transparent 96-well plate (10^5^ cells/mL). The cells were then overnight cultured and treated with BOZ (concentration range: 0.5–121 nM), TIC10 (concentration range: 0.5–121 μM) and their combinations. The used concentrations were determined based on the results of preliminary tests. After 72 h incubation, the alamarBlue^TM^ reagent was added to the wells, and then the fluorescent signal was read (excitation: 530 nm, emission: 590 nm) after 4 h by the Fluoroskan FL Microplate Fluorometer and Luminometer (Thermo Scientific, Waltham, MA, USA). In the case of the U266 myeloma cell line, it was noticed that the cell viability tests performed with alamarBlue^TM^ reagent were poorly reproducible; therefore, the CellTiter-Glo Luminescent Cell Viability Assay (Promega, Madison, WI, USA) was utilized. The cells were seeded in a white-walled 96-well plate (Thermo Scientific, Waltham, MA USA) at a density of 10^5^ cells/mL. Following overnight incubation, the cells were treated with BOZ (concentration range: 0.5–121 nM), TIC10 (concentration range: 0.5–121 μM) and their combinations. After 72 h incubation, the CellTiter-Glo Reagent was added to the wells, and the luminescence signal was read by the Fluoroskan FL Microplate Fluorometer and Luminometer (Thermo Scientific, Waltham, MA, USA). Both experiments were performed in triplicates, and the results were normalized to the untreated medium control. 

### 4.4. Synergy Quantification

To characterize the synergistic, additive or antagonistic effects of the BOZ+TIC10 combinations, the Chou–Talalay algorithm was used. With this free software, the combination index (CI) can be calculated [40] from the normalized cell viability values in the CompuSyn software (ComboSyn Inc., Paramus, NJ, US), which describes the dose–effect relationship of drugs and their combinations. Combination index < 1, = 1 or > 1 represents synergism, additive effect or antagonism, respectively. 

### 4.5. Real-Time Viability Assay

A non-invasive, impedimetric technique was utilized to validate the results of the endpoint cell viability assay in A2058 cells. The adherent cells were seeded (10^5^ cells/mL) in a special 96-well plate (E-Plate 96 PET; ACEA Biosciences, San Diego, CA, USA). The bottom of the wells is covered by gold microelectrodes that can register the changes in impedance in an electrically conductive solution. These changes are proportional to the number of cells; thus, the antiproliferative effects of the compounds can be measured. Before seeding the cells, a baseline with a cell-free culture medium was assessed. Following overnight incubation, the cells were treated with 13.5 nM BOZ + 13.5 μM TIC10, 13.5 nM BOZ + 40.5 μM TIC10 and the matching single agents and controls (medium and DMSO). The data were registered by the RTCA 2.0 software (Real Time Cell Analyzer; ACEA Biosciences, San Diego, CA, USA). This software is capable of converting the measured impedance data to a unitless cell index (cell index = (Z_ti_ − Z_t0_)/F_i_; Z_ti_: impedance at individual time point; Z_t0_: impedance at start of experiment; F_i_: constant). The experiment was performed in triplicates, and the results were normalized to the untreated medium control and represented as mean ± SD.

### 4.6. Apoptosis Assay and Brightfield Images

The translocation of phosphatidylserine to the outer membrane, an early sign of apoptosis, was investigated after staining with Annexin V (Ax V) fluorescein isothiocyanate (FITC) conjugate by flow cytometry. The membrane integrity was measured by 7-Aminoactinomycin (7-AAD). The cells were seeded in a 24-well plate at a final density of 7 × 10^4^ cells/mL. The next day, the cells were treated with the two selected combinations: 13.5 nM BOZ + 13.5 μM TIC10 and 13.5 nM BOZ + 40.5 μM TIC10, as well as the matching single agents and controls (medium and DMSO). After the 72 h incubation time, the cells were harvested with TrypLE reagent (Thermo Fisher Scientific, Waltham, MA, USA), then resuspended in 300 μL Annexin V Binding Buffer (Sony Biotechnology, Weybridge, UK) and stained with Ax V-FITC (Sony Biotechnology, Weybridge, UK) and 7-AAD (Sony Biotechnology, Weybridge, UK). The fluorescence signal was analyzed by flow cytometry (BD FACSCalibur, Becton–Dickinson, San Jose, CA, USA). The data management was performed in CellQuest Pro (Becton–Dickinson, San Jose, CA, USA) and Flowing 2.5.1. software (Turku Centre of Biotechnology, Turku, Finland). The experiment was performed in duplicates, and the results were normalized to the untreated medium control and represented as mean ± SD.

Brightfield images of the cells were taken by the Celldiscoverer 7 system using a 5× Plan-Apochromat λ/0.35 NA objective with 2× tube lens (Carl Zeiss AG, Jena, Germany) before the apoptosis assay.

### 4.7. Cytotoxicity Assay

CyQUANT^TM^ LDH Cytotoxicity Assay Kit (Invitrogen Corporation, New York, NY, USA) was used to detect cytotoxicity, as lactate dehydrogenase (LDH) release is an indicator of cell cytotoxicity [41]. Every reagent was provided in the kit. A2058 cells (7.5 × 10^5^ cells/mL) were seeded in a transparent 96-well plate. After 72 h treatment, 50 μL media of each sample was transferred to a new transparent 96-well plate. For 30 min, the wells were incubated with 50 μL Reaction Mixture, then the reaction was stopped by adding 50 μL Stop Solution to each sample well and absorbance was obtained at 490 nm by a plate reader (Multiskan MS, Labsystems, Helsinki, Finland). The experiment was performed in triplicates, and the results were normalized to the untreated medium control and represented as mean ± SD.

### 4.8. RNA Isolation and Gene Expression Analysis of Death Receptors

To investigate whether the combination treatments can influence the expression of DR4 and DR5 at the mRNA level, a quantitative reverse transcription PCR study was carried out. Total ribonucleic acid (RNA) was isolated with the RNeasy Mini Kit (Qiagen, Hilden, Germany) from treated and untreated cells according to the manufacturer’s manual. We then measured RNA concentration with a NanoDrop-1000 Spectrophotometer (Thermo Fisher Scientific, Waltham, MA, USA) instrument. The SensiFAST^TM^ cDNA Synthesis Kit (Bioline Reagents Ltd., London, UK) was used to reverse transcribe the RNA (750 ng/20 μL) to cDNA. Amplification was performed with the Sso Advanced Universal SYBR Green Supermix (Biorad, Hercules, CA, USA) in 20 μL final volume and measured by the CFX96 Touch^TM^ Real-Time PCR system with the Bio-Rad CFX Maestro software (Biorad, Hercules, CA, USA). Samples were run in triplicates for the same target. ‘No template’ controls (NTC) were included for each primer. Post-amplification melting curve analysis was performed to ensure reaction specificity. The following gene-specific pre-designed primers (Biorad, Hercules, CA, USA) were used: DR4 (TNFRSF10A, unique assay ID: qHsaCID0018590); DR5 (TNFRSF10B, unique assay ID: qHsaCED0036477); glyceraldehyde-3-phosphate dehydrogenase (GAPDH, unique assay ID: qHsaCED0038674); hypoxanthine phosphoribosyltransferase (HPRT1, unique assay ID: qHsaCID0016375). Changes in gene expression were calculated according to the 2^−(^^ΔΔCt)^ method. GAPDH and HPRT1 were used as reference genes for normalization. 

### 4.9. Flow Cytometric Detection of Death Receptors

To evaluate whether the examined treatments increase the expression of DR4 and DR5 proteins, we performed flow cytometric analysis detecting the expression of these cell surface-bound receptors. The cells (10^5^ cells/mL) were seeded in a 12-well plate. After overnight culturing and then 72 h treatment with the combinations and single compounds, the cells were harvested with TrypLE reagent (Thermo Fisher Scientific, Waltham, MA, USA) and then washed two times with PBS (0.05 M phosphate buffer saline, pH = 7.4). The supernatant was decanted, and the cells were then resuspended in 250 μL PBS. The cells were stained with 3 μL phycoerythrin (PE) conjugated isotype control (Sony Biotechnology, Weybridge, UK), anti-DR4 antibody (Sony Biotechnology, Weybridge, UK) or anti-DR5 antibody (Sony Biotechnology, Weybridge, UK). After 20 min of incubation at room temperature in the dark, the samples were centrifuged (5 min, 1200 rpm). The cells were resuspended in 300 μL PBS and were analyzed by flow cytometry (BD FACSCalibur, Becton–Dickinson, San Jose, CA, USA). The data management was performed in CellQuest Pro (Becton–Dickinson, San Jose, CA, USA) and Flowing 2.5.1. software (Turku Centre of Biotechnology, Turku, Finland). The experiment was performed in duplicates, and the results were normalized to the untreated medium control and represented as mean ± SD.

### 4.10. Statistical Analysis

Evaluation of the results was performed by using MS Excel and OriginPro 2020 (OriginLab Corporation, Northampton, MA, USA) software. Data are presented as mean ± standard deviation (SD). The data were statistically analyzed by the use of one-way analysis of variance (ANOVA) followed by Fisher’s least significant difference (Fisher’s LSD) post hoc test. Every treated sample was compared to the matching untreated medium control. The statistical significance of differences is indicated in figures as follows: x: *p* ≤ 0.05, y: *p* ≤ 0.01 and z: *p* ≤ 0.001.

## 5. Conclusions

In this paper we reported in vitro viability tests performed in two different cell lines (A2058, melanoma; U266, multiple myeloma) to define the potential synergistic activity between the proteasome inhibitor BOZ and the TRAIL-inducer TIC10.

The results of endpoint cell viability assays clearly showed synergism between BOZ and TIC10 in A2058 cells after 72 h. The results of the impedance based real time measurement and apoptosis assay have provided further evidence that TIC10 could enhance the bortezomib-induced cell death by sensitizing the cells to BOZ and inducing late apoptosis in combinations. Findings of expression studies have highlighted the role of death receptor proteins (e.g., DR5) and TRAIL protein in the development of synergism between BOZ and TIC10.

Taken together, our results support the theory that combination therapy with BOZ + TIC10 may be favorable, and thus these compounds administered together are possible promising candidates for in vivo testing in model animals.

## Figures and Tables

**Table 1:** IC_50_ values were obtained after 72 h bortezomib (BOZ) or TIC10 treatment against A2058 and U266 cells.

Drug	A2058	U266
bortezomib	23.07 ± 2.41 nM	1.45 ± 0.06 nM
TIC10	ND	0.97 ± 0.26 μM

ND: not detectable.

**Table 2:** Relative death receptor mRNA expression levels after 72 h treatment with bortezomib (BOZ) or TIC10 and their combinations. The data were normalized to the medium control.

Treatment	DR4	DR5
DMSO	0.49 ± 0.04	0.32 ± 0.04
TIC10 13.5 μM	0.08 ± 0.15	0.28 ± 0.15
TIC10 40.5 μM	0.07 ± 0.05	0.46 ± 0.08
BOZ 13.5 nM	1.20 ± 0.11	0.15 ± 0.11
BOZ 13.5 nM + TIC10 13.5 μM	1.39 ± 0.02	0.42 ± 0.02
BOZ 13.5 nM + TIC10 40.5 μM	1.56 ± 0.06	0.47 ± 0.06

## Data Availability

Data is contained within the article and supplementary materials. The data presented in this study are available on request from the corresponding author.

## Supplementary Materials

The following are available online at https://www.mdpi.com/article/10.3390/ph14080820/s1, Figure S1: Summary figure showing antiproliferative effects of bortezomib (BOZ), TIC10 and their combinations on U266 cells after 72 h incubation, Figure S2: Raw data of the real-time analysis of cell viability after bortezomib (BOZ), TIC10 and co-treatments, Figure S3: Representative images of the melanoma cells that were treated as indicated for 72 h, Table S1: The possible antiproliferative effects of the solvent dimethyl sulfoxide (DMSO) on A2058 and U266 cells after 72 h.

## Abbreviations

7-AAD	7-Aminoactinomycin
ANOVA	one-way analysis of variance
Ax V	Annexin V
BOZ	bortezomib
CI	combination index
DISC	death-inducing signaling complex
DMSO	dimethyl sulfoxide
DR4	death receptor 4
DR5	death receptor 5
ECACC	European Collection of Authenticated Cell Cultures
FITC	fluorescein isothiocyanate
Fisher’s LSD	Fisher’s least significant difference
GAPDH	glyceraldehyde-3-phosphate dehydrogenase
LDH	lactate dehydrogenase
MFI	mean fluorescence intensity
NTC	no template control
RNA	ribonucleic acid
PBS	phosphate buffer
PE	phycoerythrin
RFI	ratio of mean fluorescence intensity
RTCA	Real Time Cell Analyzer
SD	standard deviation
TIC10	TRAIL-inducing compound
TNFRSF10A	TNF Receptor Superfamily Member 10a/death receptor 4
TNFRSF10B	TNF Receptor Superfamily Member 10b/death receptor 5
TRAIL	TNF-related apoptosis-inducing ligand
UV	ultraviolet

## References

[1] Pham D.D.M., Guhan S., Tsao H. (2020). KIT and Melanoma: Biological Insights and Clinical Implications. Yonsei Med. J..

[2] Bertrand J.U., Steingrimsson E., Jouenne F., Bressac-de Paillerets B., Larue L. (2020). Melanoma Risk and Melanocyte Biology. Acta Derm. Venereol..

[3] Leonardi G.C., Falzone L., Salemi R., Zanghì A., Spandidos D.A., McCubrey J.A., Candido S., Libra M. (2018). Cutaneous melanoma: From pathogenesis to therapy (Review). Int. J. Oncol..

[4] Rastrelli M., Tropea S., Rossi C.R., Alaibac M. (2014). Melanoma: Epidemiology, Risk Factors, Pathogenesis, Diagnosis and Classification. In Vivo.

[5] Simiczyjew A., Dratkiewicz E., Mazurkiewicz J., Ziętek M., Matkowski R., Nowak D. (2020). The Influence of Tumor Microenvironment on Immune Escape of Melanoma. Int. J. Mol. Sci..

[6] Keilholz U., Ascierto P.A., Dummer R., Robert C., Lorigan P., van Akkooi A., Arance A., Blank C.U., Chiarion Sileni V., Donia M. (2020). ESMO consensus conference recommendations on the management of metastatic melanoma: Under the auspices of the ESMO Guidelines Committee. Ann. Oncol..

[7] Kozar I., Margue C., Rothengatter S., Haan C., Kreis S. (2019). Many ways to resistance: How melanoma cells evade targeted therapies. Biochim. Biophys. Acta Rev. Cancer.

[8] Steininger J., Gellrich F.F., Schulz A., Westphal D., Beissert S., Meier F. (2021). Systemic Therapy of Metastatic Melanoma: On the Road to Cure. Cancers.

[9] Gurunathan S., Kang M.H., Qasim M., Kim J.H. (2018). Nanoparticle-Mediated Combination Therapy: Two-in-One Approach for Cancer. Int. J. Mol. Sci..

[10] Takács A., Lajkó E., Láng O., Istenes I., Kőhidai L. (2020). Alpha-lipoic acid alters the antitumor effect of bortezomib in melanoma cells in vitro. Sci. Rep..

[11] Markowitz J., Luedke E.A., Grignol V.P., Hade E.M., Paul B.K., Mundy-Bosse B.L., Brooks T.R., Dao T.V., Kondalasula S.V., Lesinski G.B. (2014). A phase I trial of bortezomib and interferon-α-2b in metastatic melanoma. J. Immunother..

[12] Starobova H., Vetter I. (2017). Pathophysiology of Chemotherapy-Induced Peripheral Neuropathy. Front. Mol. Neurosci..

[13] Bahleda R., Le Deley M.C., Bernard A., Chaturvedi S., Hanley M., Poterie A., Gazzah A., Varga A., Touat M., Deutsch E. (2018). Phase I trial of bortezomib daily dose: Safety, pharmacokinetic profile, biological effects and early clinical evaluation in patients with advanced solid tumors. Investig. New Drugs.

[14] Bárány P., Oláh R.S., Kovács I., Czuczi T., Szabó C.L., Takács A., Lajkó E., Láng O., Kőhidai L., Schlosser G. (2018). Ferrocene-Containing Impiridone (ONC201) Hybrids: Synthesis, DFT Modelling, In Vitro Evaluation, and Structure—Activity Relationships. Molecules.

[15] Yuan X., Kho D., Xu J., Gajan A., Wu K., Wu G.S. (2017). ONC201 activates ER stress to inhibit the growth of triple-negative breast cancer cells. Oncotarget.

[16] Prabhu V.V., Morrow S., Rahman Kawakibi A., Zhou L., Ralff M., Ray J., Jhaveri A., Ferrarini I., Lee Y., Parker C. (2020). ONC201 and imipridones: Anti-cancer compounds with clinical efficacy. Neoplasia.

[17] Karpel-Massler G., Siegelin M.D. (2017). TIC10/ONC201-a potential therapeutic in glioblastoma. Transl. Cancer Res..

[18] Wang S., El-Deiry W.S. (2003). TRAIL and apoptosis induction by TNF-family death receptors. Oncogene.

[19] Oh Y.T., Sun S.Y. (2021). Regulation of Cancer Metastasis by TRAIL/Death Receptor Signaling. Biomolecules.

[20] Аrtykov А., Belov D.A., Shipunova V.O., Trushina D.B., Deyev S.M., Dolgikh D.A., Kirpichnikov M.P., Gasparian M.E. (2020). Chemotherapeutic Agents Sensitize Resistant Cancer Cells to the DR5-Specific Variant DR5-B more Efficiently than to TRAIL by Modulating the Surface Expression of Death and Decoy Receptors. Cancers.

[21] Liu X., Yue P., Chen S., Hu L., Lonial S., Khuri F.R., Sun S.Y. (2007). The proteasome inhibitor PS-341 (bortezomib) up-regulates DR5 expression leading to induction of apoptosis and enhancement of TRAIL-induced apoptosis despite up-regulation of c-FLIP and survivin expression in human NSCLC cells. Cancer Res..

[22] Greer Y.E., Lipkowitz S. (2015). TIC10/ONC201: A bend in the road to clinical development. Oncoscience.

[23] Pruss M., Dwucet A., Tanriover M., Hlavac M., Kast R.E., Debatin K.-M., Wirtz C.R., Halatsch M.-E., Siegelin M.D., Westhoff M.-A. (2020). Dual metabolic reprogramming by ONC201/TIC10 and 2-Deoxyglucose induces energy depletion and synergistic anti-cancer activity in glioblastoma. Br. J. Cancer.

[24] Kouroukis T.C., Baldassarre F.G., Haynes A.E., Imrie K., Reece D.E., Cheung M.C. (2014). Bortezomib in multiple myeloma: Systematic review and clinical considerations. Curr. Oncol..

[25] Manasanch E.E., Orlowski R.Z. (2017). Proteasome inhibitors in cancer therapy. Nat. Rev. Clin. Oncol..

[26] Chou T.C. (2008). Preclinical versus clinical drug combination studies. Leuk. Lymphoma.

[27] Chen X., Thibeault S. Effect of DMSO concentration, cell density and needle gauge on the viability of cryopreserved cells in three dimensional hyaluronan hydrogel. Proceedings of the Annual International Conference of the IEEE Engineering in Medicine and Biology Society (EMBC).

[28] Yan G., Du Q., Wei X., Miozzi J., Kang C., Wang J., Han X., Pan J., Xie H., Chen J. (2018). Application of Real-Time Cell Electronic Analysis System in Modern Pharmaceutical Evaluation and Analysis. Molecules.

[29] Lü S., Wang J. (2013). The resistance mechanisms of proteasome inhibitor bortezomib. Biomark. Res..

[30] Politou M., Karadimitris A., Terpos E., Kotsianidis I., Apperley J.F., Rahemtulla A. (2006). No evidence of mutations of the PSMB5 (beta-5 subunit of proteasome) in a case of myeloma with clinical resistance to Bortezomib. Leuk Res..

[31] De Wilt L.H., Jansen G., Assaraf Y.G., van Meerloo J., Cloos J., Schimmer A.D., Chan E.T., Kirk C.J., Peters G.J., Kruyt F.A. (2012). Proteasome-based mechanisms of intrinsic and acquired bortezomib resistance in non-small cell lung cancer. Biochem. Pharm..

[32] Prabhu V.V., Talekar M.K., Lulla A.R., Kline C.L.B., Zhou L., Hall J., Van den Heuvel A.P.J., Dicker D.T., Babar J., Grupp S.A. (2018). Single agent and synergistic combinatorial efficacy of first-in-class small molecule imipridone ONC201 in hematological malignancies. Cell Cycle.

[33] Ishizawa J., Kojima K., Chachad D., Ruvolo P., Ruvolo V., Jacamo R.O., Borthakur G., Mu H., Zeng Z., Tabe Y. (2016). ATF4 induction through an atypical integrated stress response to ONC201 triggers p53-independent apoptosis in hematological malignancies. Sci. Signal.

[34] Allen J.E., Krigsfeld G., Mayes P.A., Patel L., Dicker D.T., Patel A.S., Dolloff N.G., Messaris E., Scata K.A., Wang W. (2013). Dual inactivation of Akt and ERK by TIC10 signals Foxo3a nuclear translocation, TRAIL gene induction, and potent antitumor effects. Sci. Transl. Med..

[35] Allen J.E., Krigsfeld G., Patel L., Mayes P.A., Dicker D.T., Wu G.S., El-Deiry W.S. (2015). Identification of TRAIL-inducing compounds highlights small molecule ONC201/TIC10 as a unique anti-cancer agent that activates the TRAIL pathway. Mol. Cancer.

[36] Karpel-Massler G., Bâ M., Shu C., Halatsch M.E., Westhoff M.A., Bruce J.N., Canoll P., Siegelin M.D. (2015). TIC10/ONC201 synergizes with Bcl-2/Bcl-xL inhibition in glioblastoma by suppression of Mcl-1 and its binding partners in vitro and in vivo. Oncotarget.

[37] Pan G., O‘Rourke K., Chinnaiyan A.M., Gentz R., Ebner R., Ni J., Dixit V.M. (1997). The receptor for the cytotoxic ligand TRAIL. Science.

[38] Sträter J., Hinz U., Walczak H., Mechtersheimer G., Koretz K., Herfarth C., Möller P., Lehnert T. (2002). Expression of TRAIL and TRAIL receptors in colon carcinoma: TRAIL-R1 is an independent prognostic parameter. Clin. Cancer Res. Off. J. Am. Assoc. Cancer Res..

[39] Bychkov M.L., Gasparian M.E., Dolgikh D.A., Kirpichnikov M.P. (2014). Combination of TRAIL with bortezomib shifted apoptotic signaling from DR4 to DR5 death receptor by selective internalization and degradation of DR4. PLoS ONE.

[40] Chou T.-C. (2010). Drug Combination Studies and Their Synergy Quantification Using the Chou-Talalay Method. Cancer Res..

[41] Kumar P., Nagarajan A., Uchil P.D. (2018). Analysis of Cell Viability by the Lactate Dehydrogenase Assay. Cold Spring Harb. Protoc..

