# Supplementary material for: The Synergistic Activity of Bortezomib and TIC10 against A2058 Melanoma Cells
Source: Pharmaceuticals (Basel). 2021 Aug 20;14(8):820. doi: 10.3390/ph14080820 (PMC8399995; doi:10.3390/ph14080820)
Supplement: Supplementary file 1 [file pharmaceuticals-14-00820-s001.zip › pharmaceuticals-1291393-Supplementary.pdf]

pharmaceuticals

Supplementary Materials: Predicting synergism of bortezomib and TIC10 in melanoma cells

Angéla Takács, Zsófia Szász, Márton Kalabay, Péter Bárány, Antal Csámpai, Hargita Hegyesi, Orsolya Láng, Eszter Lajkó, László Kőhidai

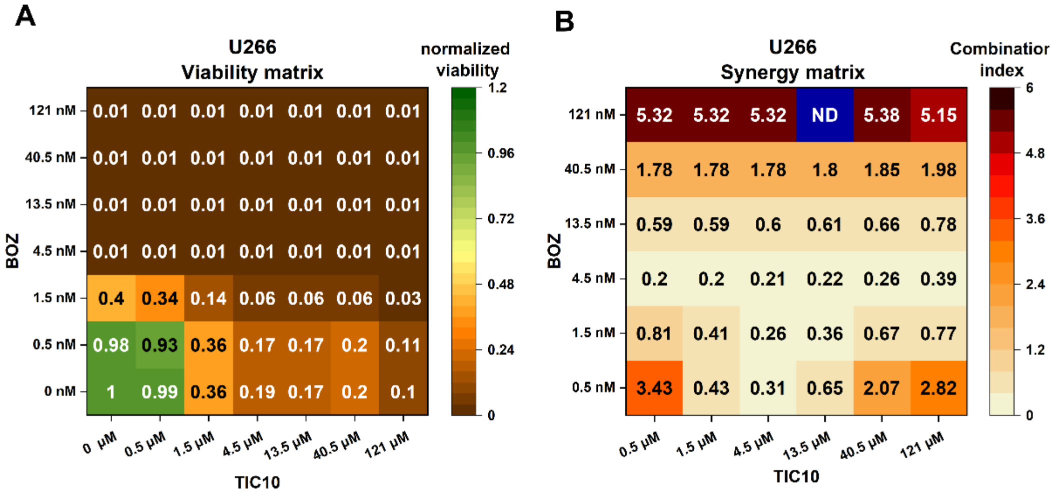

Figure S1. Summary figure showing antiproliferative effects of bortezomib (BOZ), TIC10, and their combinations on U266 cells after 72 h long incubation. (**A**) Normalized viability data are expressed as a ratio of medium control. (**B**) Combination index heat map of various drug ratios. ND: not detectable. Data are given as mean values, (n = 3).

Table S1. The possible antiproliferative effects of the solvent dimethyl sulfoxide (DMSO) on A2058 and U266 cells after 72 h. The data were normalized to the medium control. Data are given as mean values, (n=3).

DMSO (*v*/*v*%)	Normalized Cell Viability	
	A2058	U266
0.13	0.99	1.03
0.40	0.97	0.98
1.21	1.00	0.75

CitationTakács, A.; Szász, Z.; Kalabay, M.; Bárány, P.; Csámpai, A.; Hegyesi, H.; Láng, O.; Lajkó, E.; Kőhidai, L. The Synergistic Activity of Bortezomib and TIC10 against A2058 Melanoma Cells. *Pharmaceuticals* **2021**, *14*, x. https://doi.org/10.3390/ph14080820

Academic EditorDhimant Desai and Mary J. Meegan

Received: 29 June 2021
Accepted: 17 August 2021
Published: 20 August 2021

Publisher's Note: MDPI stays neutral with regard to jurisdictional claims in published maps and institutional affiliations.

Copyright: © 2021 by the authors. Submitted for possible open access publication under the terms and conditions of the Creative Commons Attribution (CC BY) license (https://creativecommons.org/licenses/by/4.0/).

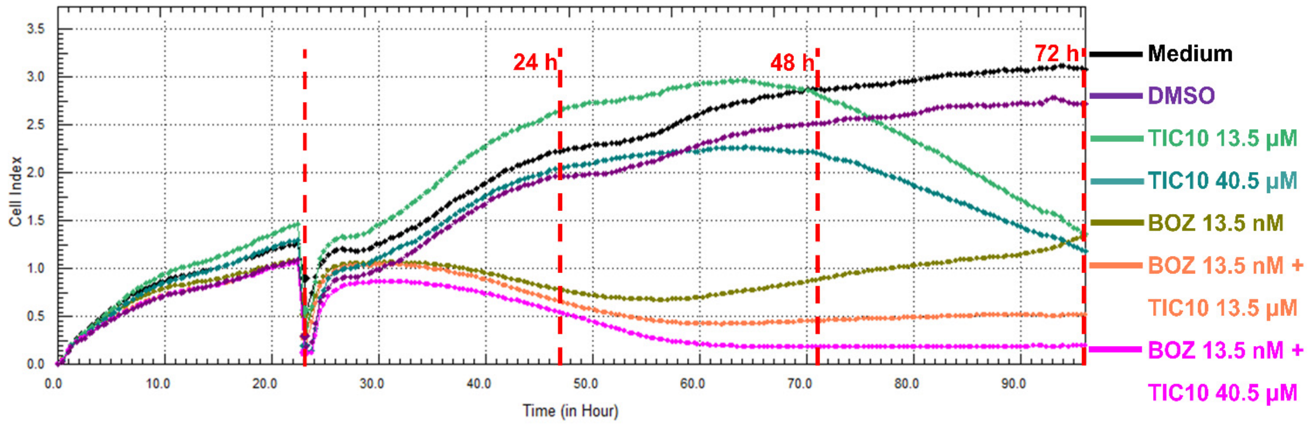

Figure S2. Raw data of the real time analysis of cell viability after bortezomib (BOZ), TIC10, and co-treatments. Cell Index, a relative and dimensionless value, represents the impedance changing due to cell adhesion, spreading (0–24 h measurement interval) and then cell viability decreasing effect of the treatments (24–96 h measurement interval). Data are represented as mean values (n = 3).

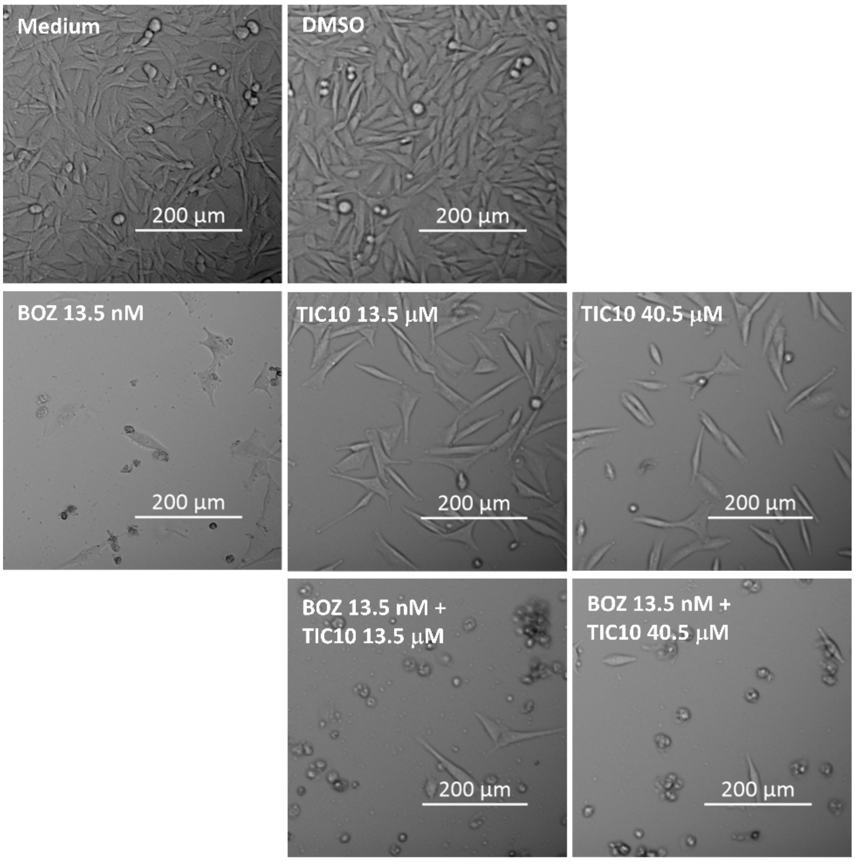

Figure S3. Representative images of the melanoma cells that were treated as indicated for 72 h. The cells were imaged on Zeiss Celldiscoverer 7 using 10-times magnification. The scale bar represents 200 µm.
